# Metabolism in Male Reproductive Aging

**DOI:** 10.20900/agmr20210005

**Published:** 2021-01-12

**Authors:** Ralph G. Meyer, Mirella L. Meyer-Ficca

**Affiliations:** 1 Department of Animal, Dairy, and Veterinary Sciences, College of Agriculture and Applied Sciences, Utah State University, Logan, UT 84332, USA; 2 Utah Experimental Station, Utah State University, Logan, UT 84332, USA; 3 School of Veterinary Medicine, Utah State University, Logan, UT 84332, USA

**Keywords:** reproductive health, aging, sperm, DNA integrity, chromatin, nicotinamide adenine dinucleotide (NAD), redox stress, testosterone, testis, developmental origin of health and disease (DOHaD)

## Abstract

Similar to female reproductive health, male reproductive health declines with increasing age, albeit in a more gradual way. In the US, the average age of first-time fathers has been steadily increasing since 1980. This is concerning because increasing paternal age is positively correlated with reduced sperm chromatin quality and higher numbers of DNA strand breaks (DNA sb), which negatively affects pregnancy outcome and child development.

While underlying reasons are not well understood, one of the well-known hallmarks of aging is a significant decline of body nicotinamide adenine dinucleotide (NAD) levels.

We propose that low body-wide NAD levels provide a plausible explanation for metabolic alterations that are associated with declining hormonal production and testicular volume, as well as reduced sperm quality in aging men.

## INTRODUCTION

In the US, average human life expectancy has increased significantly since 1900 to about 85 years between men and women, because early childhood mortality has been almost abolished. However, maximal lifespan of individual people is unlikely to increase beyond the currently observed numbers of years humans are capable of living, and current medical research has shifted to focus on increasing “healthspan” rather than lifespan, with the goal of maintaining health into old age for as long as possible [1].

Men’s healthspan is in part determined by gonadal function, which decreases in older men. This age-related decrease in gonadal function and testicular decline is commonly described as late-onset hypogonadism (LOH). LOH comprises numerous symptoms including loss of libido, erectile dysfunction, loss of muscle mass, increased visceral fat, metabolic syndrome, anemia, reduced bone density, depressed mood, decreased vitality, sweating, and hot flushes [2].

The prevailing assumption that robust fertility for a man will continue well past a woman’s decline in fertility is untrue. While the female ovarian reserve is perhaps the most crucial component of a couple’s fecundity, increasing age of the male partner is also strongly associated with increased time to conception [3]. Importantly, in addition to the overall somatic changes and the testicular decline, paternal aging results in the production of sperm with lower genetic quality, characterized by increased DNA damage and fragmentation, mutations and aneuploidies and various epigenetic changes ([4,5], reviewed e.g., in [5]). This is concerning, because the average age of first-time fathers has been steadily increasing since 1980 [6], and paternal age is positively correlated with poor pregnancy outcome and child development [7]. The mechanisms that link paternal age and deteriorating sperm quality are not well understood, but are likely due to a combination of the adverse metabolic changes that occur during the age-associated health decline.

## NAD DECLINE DURING AGING

In the context of reproductive aging, it is important to consider lower NAD levels as another important and accepted hallmark of aging in general [8,9]. NAD is a coenzyme that is found in all living cells, where it is present in various oxidative and phosphorylated states (NAD^+^, NADH, NADP^+^, NADPH). Those variants are integral coenzymes necessary for most metabolic reactions, and they are essential for the biochemical reactions that control energy metabolism pathways, including the tricarboxylic acid (TCA) cycle, oxidative phosphorylation, glycolysis, and beta-oxidation of fatty acids [10,11]. Due to its importance for all biochemical processes, NAD is regarded as a master regulator of metabolism [12].

Beyond these essential redox coenzyme functions, NAD serves as the substrate for enzymes like CD38, SARM1, sirtuins and enzymes of the poly(ADP-ribose) polymerase (PARP) family [9,13,14]. CD38 is a multifunctional protein on immune cells with roles in immune response and metabolic regulation; SARM1 is a neuronal protein involved in tissue homeostasis. Sirtuins are NAD-dependent histone-deacetylases important for chromatin remodeling and gene expression control, while PARP enzymes control DNA accessibility and DNA repair in a DNA strand break-dependent manner [12,14,15].

Aging-associated low NAD levels are characterized by a more dramatic decline of the oxidized form (NAD^+^) compared to the reduced form (NADH), which causes an imbalance of the cellular redox potential [15,16]. The resulting increase of pro-inflammatory activities is known as “inflammaging”—which is another hallmark of the aging process [17]. Elevated ROS production due to abnormal NAD redox status is further exacerbated by impaired GSH production due to the drop in NADP, as outlined further below.

In summary, the disproportional loss of NAD^+^ with increasing age has detrimental consequences on genomic and genetic integrity of cells and tissues, because the molecule has important roles in DNA repair and chromatin regulation, and it is important for maintaining an appropriate redox potential in the cell. Low NAD^+^ levels therefore present a reasonable explanation for the observed increased oxidative stress and weakened DNA repair capacities of aging tissues.

## NAD DECLINE AND REDOX BALANCE DURING TESTICULAR AGING

Testicular volume begins to become lower in males over the age of 60 years, and in men older than 75 years, testis volumes are on average less than 70% of those typical of young men [18].

Age-related alterations, such as a changing hypothalamus-pituitary hormonal axis and a reduced ability of Leydig cells to maintain serum testosterone levels, contribute to this testicular decline [19]. Concomitant with slower testosterone synthesis, elderly men, who are otherwise healthy, have increased serum levels of hormones that stimulate testosterone synthesis, such as LH and FSH. Along the same line, testosterone metabolites like estradiol and inhibin, a factor involved in the negative feedback loop controlling testosterone synthesis, are significantly lowered [18,20]. While the molecular mechanisms that cause the age-related Leydig cell defects are still not fully understood, there is mounting evidence that hypothalamic functions are regulated through NAD and sirtuins, and that aging-related NAD decline causes deregulation of the hypothalamus [21]. One additional explanation may be provided by an age-associated increase in oxidative stress, i.e., an imbalance in the cellular redox systems that results in a net increase of pro-oxidants and a decrease of antioxidants [22]. This imbalance ultimately leads to the accumulation of reactive oxygen species (ROS) that functionally impair multiple cellular biochemical processes, including steroidogenesis, through damaging DNA, proteins and lipids. The redox imbalance results from either increased radical formation, or from decreased defense mechanisms against radicals, or both. For example, progressive age-related mitochondrial dysfunction has been proposed as one of the reasons for increased radical formation in the cell that ultimately causes age-related testicular dysfunction and degenerative disease [23].

At the same time, a decline in cellular antioxidant activity of cellular ROS scavenging systems, such as superoxide dismutases, catalase, thioredoxins, peroxiredoxins and glutathione (GSH) may contribute to the altered redox environment in the aging testis. GSH, a sulfhydryl containing tripeptide γ-glutamyl-cysteinylglycine, is an important example of such an intracellular antioxidant in cells. GSH, a sulfhydryl containing tripeptide γ-glutamyl-cysteinylglycine, is one important intracellular antioxidant in cells—it has even been considered “the master antioxidant”—whose general abundance is known to decrease with age [24,25]. A healthy balance between the active, reduced glutathione form GSH and its inactive oxidized form, GSSG, is preserved by the enzyme glutathione reductase, which maintains high levels of active GSH that are able to detoxify harmful reactive oxygen species (ROS). The enzymatic activity of glutathione reductase directly depends on cellular levels of NADPH. NADPH is created from NAD by the activity of NAD kinase, and NADPH levels, like NAD levels, are lower in the aging individual [26].

The age-related NAD deficiency will therefore weaken cellular defenses against ROS by limiting GSH production and increase oxidative stress in the testis, contributing to an imbalance of cellular redox potential. The resulting increased ROS levels have the potential to negatively affect DNA integrity, healthy germ cell development, and impair the steroidogenesis pathway in Leydig cells, which results in a progressive decline in testosterone synthesis and hypogonadism [27].

## AGING AND SPERM QUALITY

During their differentiation, spermatids, which are the post-meiotic haploid male germ cells, undergo extensive nuclear and chromatin remodeling processes. Those changes are necessary for the generation of fertilization-competent healthy sperm with the proper DNA integrity and epigenetic information to ultimately support fertilization and development of healthy offspring. The process of sperm chromatin reorganization requires the transient and controlled formation of numerous endogenous DNA strand breaks [28,29], as well as efficient removal of histones and their replacement by sperm-specific nuclear proteins called protamines [30]. Both chromatin remodeling processes are controlled by an interplay of many chromatin-modifying enzymes, including PARPs and NAD-dependent histone deacetylases [31]. Impaired PARP and sirtuin activities in animal models with gene deletions resulted in reduced sperm chromatin quality and function [32–35]. Decelerated functionality of these enzymes, due to worsening NAD deficiency in the aging male, may therefore provide some additional explanation why sperm quality in older men is inferior to sperm quality in younger men [36,37]. In line with these considerations, semen of men of advanced age has increased levels of ROS [5,37]. Their sperm nuclei tend to contain immature chromatin with more accumulated DNA damage, seen as sperm DNA oxidation and fragmentation [5,38]. ROS also target sterols and polyunsaturated lipid molecules in the sperm membrane. This lipid peroxidation results in the formation of various cytotoxic byproducts and importantly interferes with the normal fluidity of sperm membranes, which in necessary for sperm motility, sperm capacitation and ultimately sperm-egg fusion [39–42]. All those consequences of excessive ROS exposure ultimately contribute to the reduced fertility of older men.

## REPRODUCTIVE AGING, EPIGENETICS, AND OFFSPRING HEALTH

While women over 35 years of age have long been known to incur an increased risk of giving birth to babies with chromosomal abnormalities, such as trisomy 21, male age has not been widely recognized as a determining factor for safe procreation. This view is changing, and based on statistical evaluation of clinical data, the age of 40 has been recommended by a study as the cutoff for assisted reproductive technique attempts [4].

Besides the reduced fertility noted in older men, the diminished sperm chromatin quality holds increased risks for offspring health [43]. Poor sperm genetic integrity, with DNA strand breaks (DNA fragmentation) and poor chromatin maturation characterized by excessive retention of histones and insufficient protamine deposition, are now widely recognized as male-factor causes of abnormal embryonic development and recurrent pregnancy loss [44,45]. Among the most dramatic adverse effect of advanced paternal age is an increased risk for stillbirth [46]. Other negative consequences are increased frequencies of congenital defects and genetic diseases in children born to older fathers, underpinning the importance of sperm chromatin- and DNA-integrity.

In addition to these strictly genetic defects, seemingly indirect consequences of advanced paternal age, such as incidences of cancer and mental diseases, including schizophrenia and autism, are also increased in these progeny [7,47]. The direct molecular links in the latter cases are not well understood, but likely are connected to altered epigenetic information in sperm of father with advanced age [48–50]. Epidemiological observations provide a large body of evidence that paternal epigenetic inheritance is an important factor of metabolic programming in humans [51–54]. Epigenetic inheritance through the male germ line may, for instance, contribute to the current epidemic of obesity and metabolic disease in the US [53,55–58], where the impact of paternal age remains unclear. However, paternal age is positively correlated with a healthy body mass index in offspring, but unfortunately it also promotes offspring dyslipidemia [59]. In line with the paradigm of developmental origin of health and disease (DOHaD), the term “Paternal Origin of Health and Disease” (POHaD) has recently been coined to designate the growing field of research in this area [60]. Besides DNA methylation and various non coding RNAs, sperm chromatin has been shown to be a carrier of epigenetic information [31,61,62]. Since epigenetic signatures in sperm rely, among other pathways, on the activity of PARP and sirtuin enzymes, the age-related NAD decline likely contributes to both, the declining genomic integrity and the inferior epigenetic signatures in sperm of aging men.

## SUMMARY

The decline in male reproductive fitness that occurs with advancing age comprises direct effects, including impaired hormonal homeostasis and decreasing testicular volume, as well as deteriorating sperm quality, fertility problems and, ultimately, impaired offspring health.

**Figure 1.**
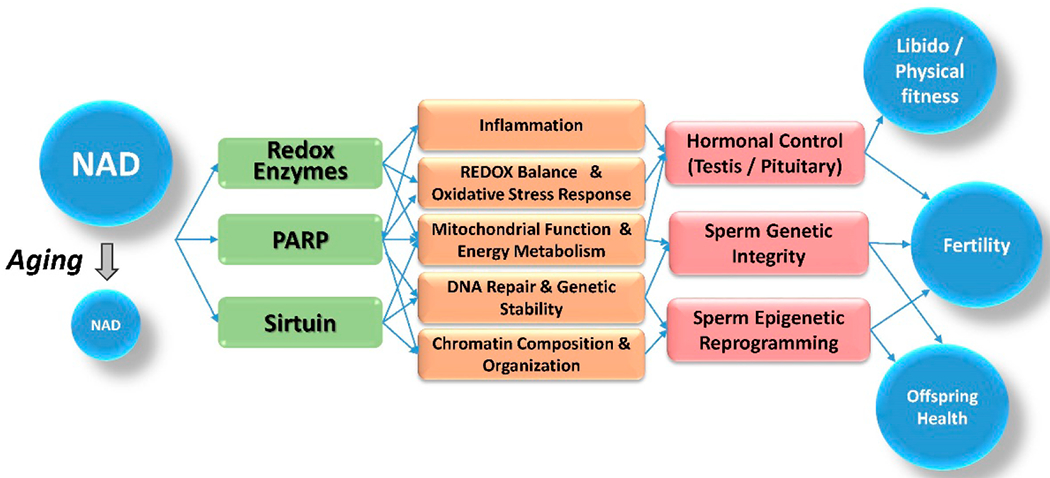
Link between NAD and reproductive health. The availability of NAD determines how active cellular redox enzymes and epigenetic modifiers like PARPs and sirtuins can be, which in turn regulates cellular redox balance, DNA repair capability, chromatin organization and inflammation. In the context of reproductive health, declining NAD availability during aging thus can negatively affect hormonal control and germ cell quality, and ultimately results in diminished reproductive fitness and offspring health.

The symptoms of reproductive aging in males share common molecular pathways and regulators that depend on a precise control of the cellular redox balance and energy metabolism. A central player in these molecular processes is the essential metabolic cofactor NAD. One of the hallmarks of aging is a marked decrease of cellular NAD levels over time, which results in a shift in the cellular redox balance. An increasing body of evidence therefore suggests that failing NAD levels provide a plausible, overarching link between different aspects of aging, such as metabolic disease leading to increased inflammation, reduced testicular function, poor sperm quality and compromised offspring health (Figure 1).
